# Membrane lipids in *Agrobacterium tumefaciens*: biosynthetic pathways and importance for pathogenesis

**DOI:** 10.3389/fpls.2014.00109

**Published:** 2014-03-26

**Authors:** Meriyem Aktas, Linna Danne, Philip Möller, Franz Narberhaus

**Affiliations:** Microbial Biology, Department for Biology and Biotechnology, Ruhr University Bochum*Bochum, Germany*

**Keywords:** membrane lipids, phospholipid biosynthesis, phosphatidylcholine, phosphorus-free lipids, ornithine lipids, glycolipids, betaine lipids, *Agrobacterium tumefaciens*

## Abstract

Many cellular processes critically depend on the membrane composition. In this review, we focus on the biosynthesis and physiological roles of membrane lipids in the plant pathogen *Agrobacterium tumefaciens*. The major components of *A. tumefaciens* membranes are the phospholipids (PLs), phosphatidylethanolamine (PE), phosphatidylglycerol, phosphatidylcholine (PC) and cardiolipin, and ornithine lipids (OLs). Under phosphate-limited conditions, the membrane composition shifts to phosphate-free lipids like glycolipids, OLs and a betaine lipid. Remarkably, PC and OLs have opposing effects on virulence of *A. tumefaciens*. OL-lacking *A. tumefaciens* mutants form tumors on the host plant earlier than the wild type suggesting a reduced host defense response in the absence of OLs. In contrast, *A. tumefaciens* is compromised in tumor formation in the absence of PC. In general, PC is a rare component of bacterial membranes but amount to ~22% of all PLs in *A. tumefaciens*. PC biosynthesis occurs via two pathways. The phospholipid *N*-methyltransferase PmtA methylates PE via the intermediates monomethyl-PE and dimethyl-PE to PC. In the second pathway, the membrane-integral enzyme PC synthase (Pcs) condenses choline with CDP-diacylglycerol to PC. Apart from the virulence defect, PC-deficient *A. tumefaciens* *pmtA* and *pcs* double mutants show reduced motility, enhanced biofilm formation and increased sensitivity towards detergent and thermal stress. In summary, there is cumulative evidence that the membrane lipid composition of *A. tumefaciens* is critical for agrobacterial physiology and tumor formation.

## INTRODUCTION

**FIGURE 1:**
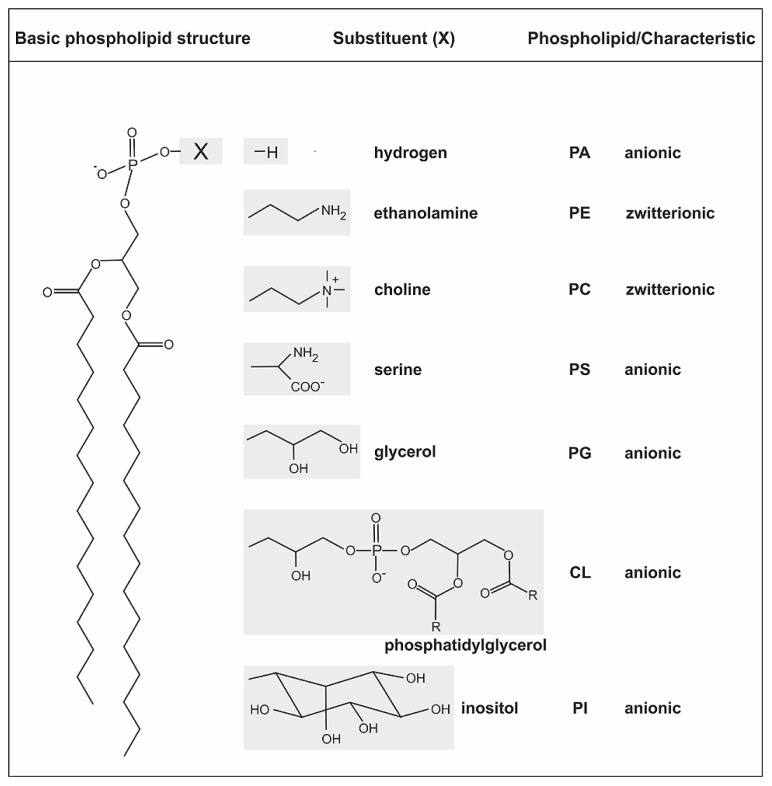
**General structure of phospholipids and common head groups.** PLs contain two fatty acids ester-linked to glycerol at C-1 and C-2, and a polar head group attached at C-3 via a phosphodiester bond. The fatty acids in PLs can vary in carbon group length and saturation degree. The different common polar head groups and charges are indicated. PA, phosphatidic acid; PE, phosphatidylethanolamine; PC, phosphatidylcholine; PS, phosphatidylserine; PG, phosphatidylglycerol; CL, cardiolipin; PI, phosphatidylinositol.

The structure of biological membranes is mainly defined by heterogeneous amphipathic phospholipids (PLs) forming the phospholipid bilayer. PLs contain a diacylglycerol (DAG) as hydrophobic component with saturated or unsaturated fatty acyl chains of variable length and a polar head group attached to the phosphate group (Korn, 1966; van Meer et al., 2008; Wolf and Quinn, 2008). The general structure of PLs and common head groups are shown in **Figure 1**. Phosphatidylethanolamine (PE) and phosphatidylcholine (PC) are zwitterionic lipids whereas phosphatidic acid (PA), phosphatidylglycerol (PG), cardiolipin (CL), phosphatidylserine (PS), and phosphatidylinositol (PI) represent the anionic lipid class. Contrary to previous assumptions based on the fluid mosaic model (Singer and Nicolson, 1972), the lipid distribution in pro- and eukaryotic membranes is dynamic and asymmetric (Fadeel and Xue, 2009; Clifton et al., 2013). Specialized lipid micro domains (in eukaryotes referred to as lipid rafts) serve as platform for various cellular processes such as signal transduction and transport (Edidin, 2003; Zhang et al., 2005; Pike, 2006; Donovan and Bramkamp, 2009; Lingwood and Simons, 2010; LaRocca et al., 2013).

All biological membranes share the same basic membrane structure but the lipid composition differs tremendously between the domains of life and even within a domain. The lipid repertoire of eukaryotic cells is very complex. Combination of different head groups and variations in fatty acid tails results in more than a thousand different lipids. The major lipids in eukaryotes are PLs with PC as the most abundant, followed by PE, PS, PI, and PA (van Meer et al., 2008). PG is also present in eukaryotes and is used as precursor for CL synthesis, exclusively found in mitochondria (Bligny and Douce, 1980). Further important constituents of eukaryotic membranes are sphingolipids (SLs) and cholesterol, which are enriched in lipid rafts (Lingwood and Simons, 2010; Sonnino and Prinetti, 2013).

Bacterial membrane lipids are more diverse than previously thought (Parsons and Rock, 2013). Most bacteria, like the Gram-negative model organism *Escherichia coli* have a simple membrane lipid composition with the major PLs PE, PG, and CL (Ames, 1968; Cronan, 2003; López-Lara et al., 2003; Dowhan, 2009). However, many other bacteria are known to produce additional and uncommon lipids. PS is abundant in eukaryotic membranes but most prokaryotes contain only minor PS amounts as it serves as precursor for PE biosynthesis (Bunn and Elkan, 1971; López-Lara et al., 2003). Although PI is a rare component of bacterial membranes it is a major lipid in *Mycobacterium tuberculosis* where it is essential for viability (Jackson et al., 2000). SLs have been described in *Sphingobacterium, Sphingomonas,* and *Bacteroides* species (Heung et al., 2006). *Sphingomonas paucomobilis* contains two glyco-SLs in its outer membrane important for pathogenesis (Kinjo et al., 2005; Mattner et al., 2005). Some bacteria such as *Methylococcus capsulatus* or *Rhodopseudomonas palustris* TIE-1 can also synthesize steroid lipids and/or sterol homologues (hopanoid lipids; Tippelt et al., 1998; Bode et al., 2003; Doughty et al., 2011). The membrane of the Gram-positive model organism *Bacillus subtilis* comprises lysyl-PG (LPG) and up to 40% neutral glycolipids (GLs; Salzberg and Helmann, 2008). In some bacteria such as *Agrobacterium tumefaciens*, *Sinorhizobium meliloti,* and *Rhodobacter spaeroides* phosphate limitation stimulates the production of phosphate-free lipids including ornithine lipids (OLs), sulfolipids, betaine lipids, and GLs (López-Lara et al., 2003, 2005; Vences-Guzmán et al., 2012; Geske et al., 2013; Parsons and Rock, 2013). The major eukaryotic membrane lipid PC is not widespread in bacteria. It has been estimated that ~15% of all bacterial species produce PC (Sohlenkamp et al., 2003; Aktas et al., 2010; Geiger et al., 2013). It is frequently found in symbionts or pathogens and in bacteria with extensive intracytoplasmic membranes (Hagen et al., 1966; Goldfine, 1984; Geiger et al., 2013). Often, PC is critical for bacteria–host interactions.

## COMMON METABOLIC PATHWAYS FOR PHOSPHOLIPIDS IN BACTERIA

**FIGURE 2:**
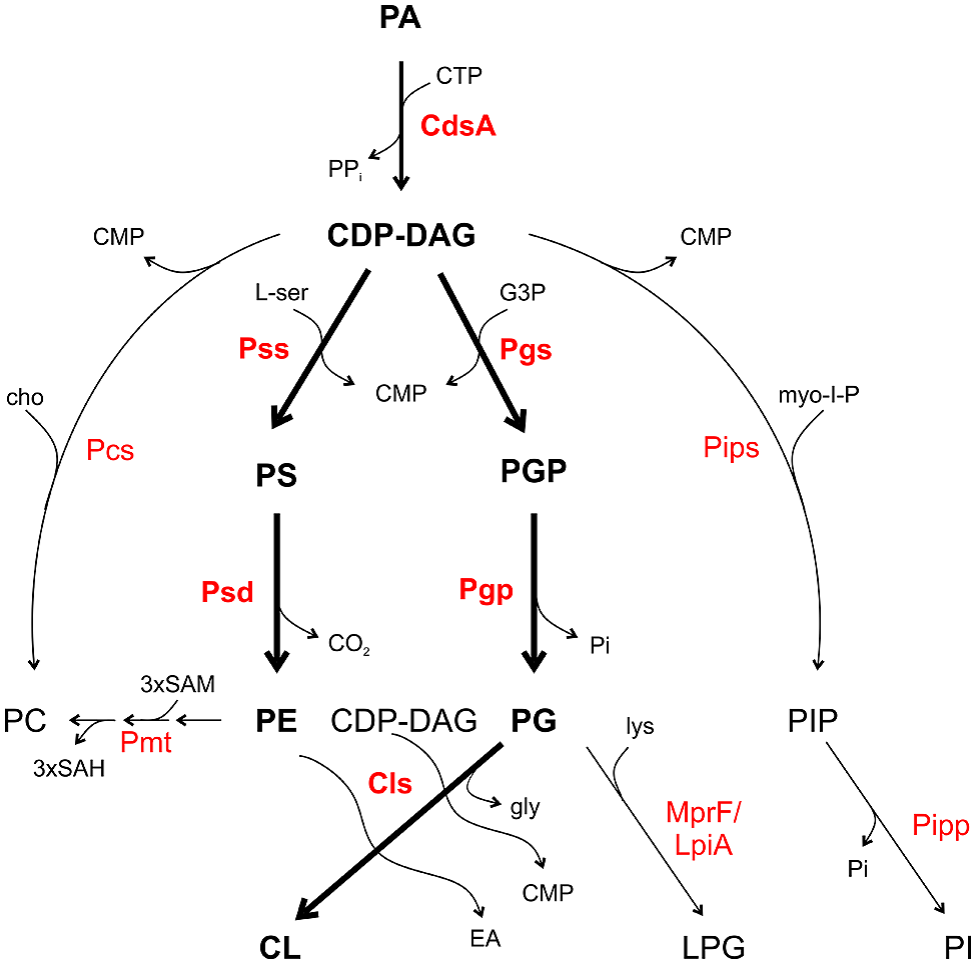
**Phospholipid pathways and enzymes in bacteria.** CDP-DAG is the central precursor for synthesis of the PLs. Thick arrows and boldface letters indicate the most common pathways and enzymes in bacteria. For details see text. CMP, cytidine monophosphate; CTP, cytidine triphosphate; EA, ethanolamine; cho, choline; G3P, glycerol 3-phosphate; gly, glycerol; lys, lysine; L-ser, L-serine; myo-I-P, myo-inositol 1-phosphate; SAM, *S*-adenosylmethionine; SAH, *S*-adenosylhomocysteine.

All major PLs in bacteria are formed from a common precursor, namely cytidine diphosphate diacylglycerol (CDP-DAG) generated by a CDP-DAG synthase (CdsA) using PA and cytidine triphosphate (CTP; **Figure 2**; Zhang and Rock, 2008; Parsons and Rock, 2013). CDP-DAG can be directly converted to PS, PG phosphate (PGP) or in some bacteria to PI phosphate (PIP) and PC. These reactions are catalyzed by specific CDP-alcohol phosphatidyltransferases releasing a CMP molecule from CDP-DAG and transferring the phosphatidyl moiety to different polar head groups (Sohlenkamp et al., 2003; Parsons and Rock, 2013). PS synthases (Pss) use L-serine as the phosphatidyl acceptor to generate the anionic lipid PS, which serves as precursor for PE synthesis via PS decarboxylases (Psd). In *mycobacteria*, a PIP synthase (Pips) converts CDP-DAG and myo-inositol 1-phosphate to PIP which is dephosphorylated via a PIP phosphatase (Pipp) to PI (Morii et al., 2010; Morii et al., 2014). PG synthases (Pgs) transfer the phosphatidyl group from CDP-DAG to a glycerol-3-phosphate (G3P) resulting in PGP, which serves as precursor for PG synthesis by PGP phosphatases (Pgp). Two PG molecules are condensed via a cardiolipin synthase (Cls) to CL. Most bacteria possess more than one Cls. *E. coli* encodes three Cls with distinct specificities. ClsA uses two PG molecules for CL formation whereas ClsC condenses a PE and PG molecule to CL. Like the other Cls enzymes, ClsB utilizes PG but the second substrate is unknown (Pluschke et al., 1978; Nishijima et al., 1988; Guo and Tropp, 2000; Tan et al., 2012). In *Streptomyces coelicolor* a eukaryotic-type Cls using CDP-DAG and PG for CL synthesis was identified. This enzyme belongs to the CDP-alcohol phosphatidyltransferase family and seems to be common in actinobacteria (Sandoval-Calderón et al., 2009).

In several Gram-positive bacteria such as *Staphylococcus aureus* and *Bacillus subtilis*, PG is converted to the positively charged lipid LPG by aminoacylation using lysyl-tRNA as the lysine donor by the Mprf (multiple peptide resistant factor) enzyme (Ernst and Peschel, 2011). In *Staphylococcus aureus*, LPG confers resistance towards cationic antimicrobial peptides (CAMPs) by perturbation of the electrostatic attraction of CAMPs (Kilelee et al., 2010; Andrä et al., 2011). MprF homologs namely LpiA (low pH inducible A) are also present in some Gram-negative bacteria such as *Rhizobium tropici* and *Sinorhizobium medicae* and confer tolerance to acid stress and selected cationic peptides (Reeve et al., 2006; Sohlenkamp et al., 2007).

Two common PC synthesis pathways operate in bacteria: the PE-methylation pathway and the PC synthase (Pcs) route. Several bacteria contain both PC synthesis pathways such as *A. tumefaciens* and* S. meliloti*. However, some species like *Rhodobacter sphaeroides* or *Zymomonas mobilis* only have the methylation pathway for PC synthesis. Some important pathogens including *Borrelia burgdorferi*, *Brucella abortus,* or *Pseudomonas aeruginosa* only possess the Pcs pathway (Martínez-Morales et al., 2003; Sohlenkamp et al., 2003; Aktas et al., 2010; Geiger et al., 2013). In the methylation pathway, one or several phospholipid *N*-methyltransferase (Pmt) enzymes transfer a methyl group from *S*-adenosylmethionine (SAM) to the amino group of PE generating the intermediates monomethyl-PE (MMPE) and dimethyl-PE (DMPE) and finally PC (**Figure 2**). The methyldonor SAM is converted to *S*-adenosylhomocysteine (SAH) during this reaction. In the bacteria-specific Pcs pathway, choline is condensed with CDP-DAG to PC releasing a CMP molecule (Sohlenkamp et al., 2000; Aktas et al., 2010; Solís-Oviedo et al., 2012; Geiger et al., 2013).

A eukaryotic-like CDP-choline pathway has been postulated in *Treponema denticola* (Kent et al., 2004) and might be also present in other *Treponema* species. This pathway involves a choline kinase (LicA) generating choline phosphate which serves as substrate for a CTP: phosphocholine cytidylyl transferase (LicC) to produce CDP-choline. In the final step, PC is formed by transferring the phosphocholine moiety to DAG by a CDP-choline transferase (CPT; Kent et al., 2004; Geiger et al., 2013).

Recently, a new PC biosynthesis route was discovered in *Xanthomonas campestris*, which produces PC via a yeast-like two-step acylation of the precursor glycerophosphocholine (Moser et al., 2014) demonstrating that quite different strategies acting on the head or tail group have evolved for PC synthesis in bacteria.

Following this general information, the remainder of this review will present an overview of biosynthetic pathways and enzymes for membrane lipids in the plant pathogen *A*.* tumefaciens* and discuss the physiological relevance of those lipids in this organism.

## MEMBRANE LIPID REPERTOIRE AND PHOSPHOLIPID BIOSYNTHESIS ENZYMES IN *A. tumefaciens*

**FIGURE 3:**
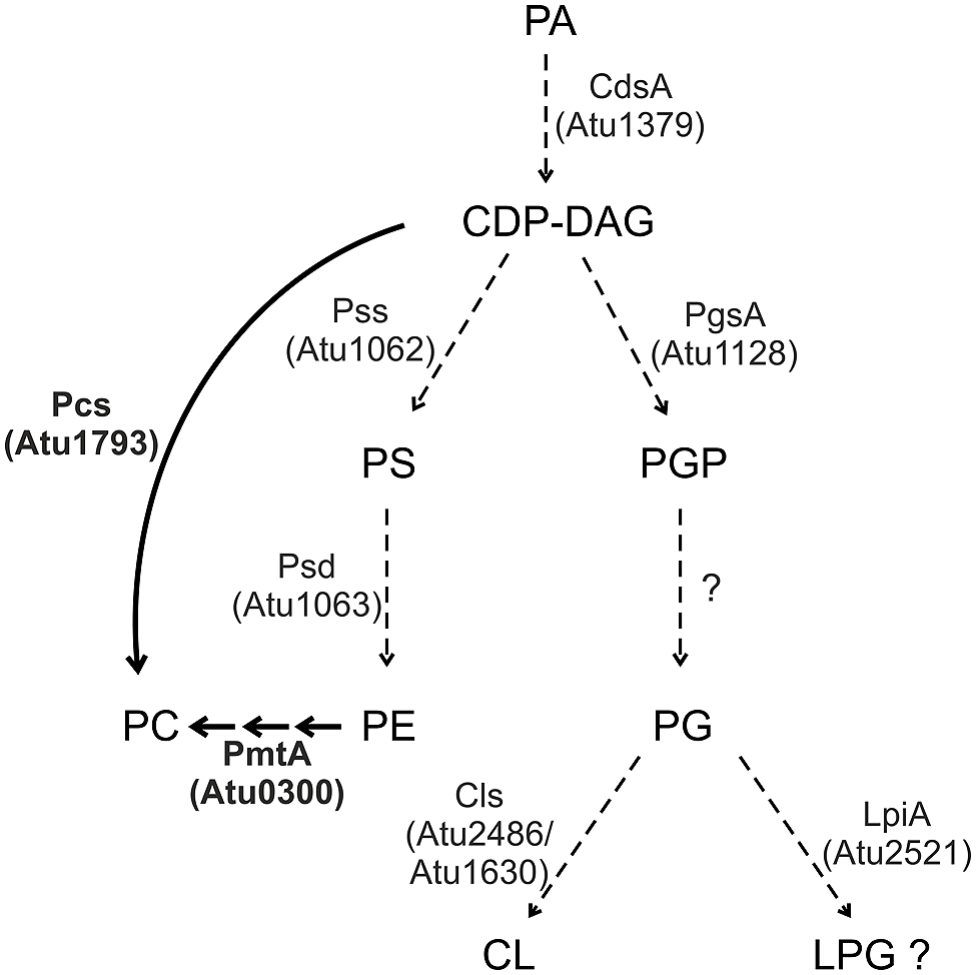
**Phospholipid synthesis in *A. tumefaciens.*** Characterized pathways are indicated by thick arrows and enzymes in boldface letters. Dashed arrows indicate the putative pathways. For details see text.

*Agrobacterium* membranes contain a rich setup of polar lipids (Randle et al., 1969; Das et al., 1979; Thompson et al., 1983; Vences-Guzmán et al., 2013; Moser et al., 2014). The lipid repertoire of several *Agrobacterium* strains has been quantified. Under full nutrition, *A. tumefaciens* membranes are mainly composed of the PLs PE and PG (account together ~45%), PC (~22%), CL (~15%), MMPE (~15%) and traces of DMPE (~4%; Moser et al., 2014). Two-dimensional thin layer chromatography and mass spectrometry analysis revealed that *A. tumefaciens* membranes also contain two OLs (Geske et al., 2013; Vences-Guzmán et al., 2013). A broad variety of membrane lipids in this organism is reflected by a lysine-containing lipid with a backbone structure similar to OLs (Tahara et al., 1976). Most of the PL synthesis pathways and enzymes in *Agrobacterium,* except for PC synthesis, are still uncharacterised. However, with the exception of a *pgp* gene, homologs for all common PL biosynthesis genes described above are encoded in the *A. tumefaciens* genome (**Figure 3**; Wood et al., 2001).

The putative *A. tumefaciens* *pss* (*atu1062*) gene is homologous to the *pss* gene from the non-pathogenic, high beta-1,3-glucan (curdlan) producing *Agrobacterium* sp. ATCC31749 (Karnezis et al., 2002; Ruffing et al., 2011). Functional analysis of its recombinant Pss protein in *E. coli* demonstrated a Mn^2^^+^-dependent [^3^H]serine incorporation into a chloroform-soluble product, most likely PS. Localisation studies in *E. coli* and topology predictions suggest that Pss is an integral membrane protein of ~30 kDa with eight transmembrane domains (TM). A cytosolic loop connecting the second and third TM contains a conserved motif (DX_2_DGX_2_ARX_5_S/TX_2_GX_3_DSX_2_D) characteristic for amino alcohol phosphatidyltransferases and thought to be involved in catalysis. A *pss* mutant is unable to produce PE suggesting that PE synthesis exclusively occurs via decarboxylation of PS. Loss of PE seems to be compensated by increased PG and CL levels in the *pss* mutant. Interestingly, the PE-deficient mutant is dramatically reduced in curdlan production and grows poorly in minimal medium. This growth defect can be compensated by Mg^2^^+^ ions, which presumably stabilize the membrane. However, curdlan production of the mutant strain cannot be cured by Mg^2^^+^. PE seems to be required for proper assembly and function of the integral inner membrane protein curdlan synthase as shown for several other membrane proteins (Karnezis et al., 2002, 2003; Raja, 2011; Bogdanov and Dowhan, 2012).

*A. tumefaciens* codes for a putative LPG synthase (Atu2521, LpiA) but LPG has not yet been identified in this organism. LPG is a major lipid in some Gram-positive bacteria but only low levels are formed in Gram-negatives. Transcription of the related* lpiA* gene in *S. medicae* is activated at low pH and is required for survival during acid stress. However, LPG was not detected even under acidic conditions in this organism suggesting production of very small amounts or rapid turnover of LPG (Reeve et al., 2006). Small amounts of LpiA-produced LPG were detected in *R. tropici* CIAT899 (~1% of the total lipids) in low pH minimal medium. Here, LPG confers resistance against the cationic peptide polymyxin B under acidic growth conditions (Sohlenkamp et al., 2007). Interestingly, *lpiA*/*mprF* homologs are present in many bacteria interacting with eukaryotes such as symbionts, pathogens and commensals suggesting that LPG might be important for bacteria–host interactions (Vinuesa et al., 2003; Sohlenkamp et al., 2007). Since low pH is one of the signals inducing virulence factors in *A. tumefaciens*, it will be of great interest to determine whether *lpiA* contributes to *Agrobacterium* pathogenesis.

### THE METHYLATION PATHWAY IN *A. tumefaciens*

**FIGURE 4:**
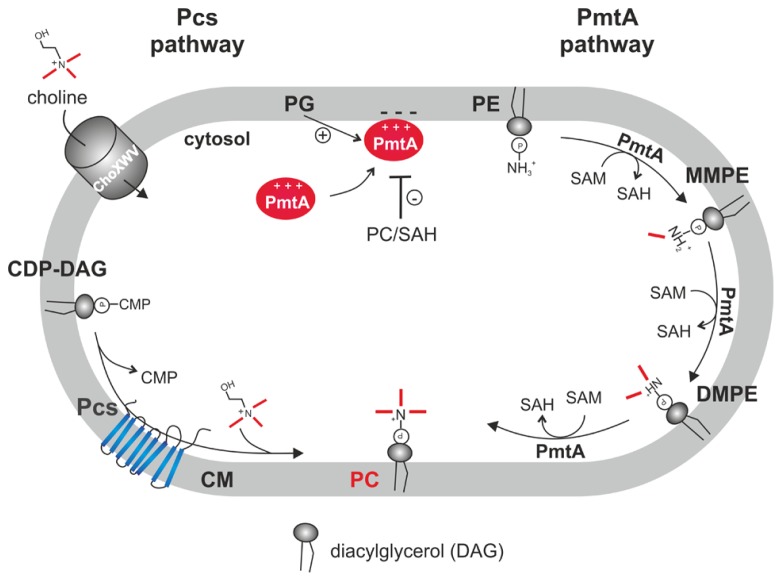
**Phosphatidylcholine biosynthesis in *A. tumefaciens.*** In the PC synthase pathway, the integral membrane protein Pcs condenses CDP-DAG and choline to PC. Choline is taken up via the ChoXWV transporter. In the PmtA pathway, a single peripheral phospholipid *N*-methyltransferase (PmtA) converts PE via three successive methylations to PC. PmtA is stimulated by the anionic lipid PG and inhibited by PC and SAH. CM, cytoplasmic membrane.

The two PC biosynthesis pathways and corresponding enzymes (Pcs and PmtA) in *A. tumefaciens* have been well characterized (**Figure 3**). Initial work on PC synthesis in *Agrobacterium* demonstrated incorporation of the ^14^C-methyl moiety of SAM into MMPE, DMPE, and PC and ^14^C-choline uptake and incorporation into PC (Kaneshiro and Law, 1964; Sherr and Law, 1965; Kaneshiro, 1968). In earlier studies, two distinct Pmts were postulated in *A. tumefaciens*. A soluble Pmt catalyzing MMPE formation only and a Pmt associated with the particulate cell fraction producing all methylated PE-derivatives (Kaneshiro and Law, 1964). The *A. tumefaciens* genome, however, contains only a single constitutively expressed* pmt* gene (*pmtA*, *atu0300*) on the circular chromosome (Wessel et al., 2006; Klüsener et al., 2009). The lack of MMPE, DMPE, and PC in a *pmtA* mutant grown without choline demonstrated that PmtA is the only enzyme responsible for MMPE, DMPE, and PC synthesis via the methylation pathway (Wessel et al., 2006). Purification of recombinant PmtA from the soluble cell fraction suggests that it is a peripheral membrane protein reversibly attaching to its site of action, the membrane (Aktas and Narberhaus, 2009; Aktas et al., 2011a). PmtA is a monomeric small enzyme (~22 kDa) catalyzing the methylation of PE to MMPE, DMPE, and PC. *In vitro* lipid binding experiments with PmtA revealed strong binding to the anionic lipids PI and PG. Interestingly, overall PmtA activity is stimulated by PG. Association of peripheral proteins with membranes is often mediated via electrostatic interactions with negatively charged PLs such as PG and a similar mechanism is proposed for the *A. tumefaciens* PmtA enzyme (**Figure 4**). SAM binding by PmtA occurs only in the presence of its substrates PE, MMPE, DMPE or the end product PC. PG alone does not influence SAM binding suggesting that two distinct binding sites for its substrates or products and for PG exist (Aktas and Narberhaus, 2009).

*In vitro* PmtA activity is negatively regulated by the end products SAH (via interfering with SAM binding) and by PC. End product-mediated inhibition might also be relevant *in vivo* to balance proper lipid composition (Aktas and Narberhaus, 2009). Like all Pmt enzymes, PmtA contains a highly conserved N-terminal SAM binding motif [VL(E/D)XGXGXG] (Sohlenkamp et al., 2003). Within this motif, the amino acids E58, G60, G62, and E84 were found to be essential for activity and SAM binding (Aktas et al., 2011a). *A. tumefaciens* PmtA seems to follow an ordered Bi–Bi reaction mechanism with initial substrate binding followed by a conformational change allowing SAM binding. Subsequently, the methyl group might be transferred to the lipid substrate releasing the first product SAH followed by the release of the methylated lipid product (Aktas et al., 2010).

Bacterial Pmts are classified into *Sinorhizobium* and *Rhodobacter* type enzymes. Enzymes belonging to the *Sinorhizobium* family including *A. tumefaciens* PmtA, are homologous to rRNA methylases, whereas *Rhodobacter*-like Pmt enzymes are similar to UbiE, ubiquinone/menaquinone biosynthesis methyltransferases. Similarities between these two Pmt families are restricted to the conserved SAM-binding motif (Sohlenkamp et al., 2003; Aktas et al., 2010; Geiger et al., 2013). The product spectrum of Pmt enzymes varies in different organisms. While *A. tumefaciens* and *S. meliloti* *pmtA* release small amounts of the intermediates MMPE and DMPE, expression of *R. sphaeroides* *pmtA* in *E. coli* exclusively resulted in PC formation (Arondel et al., 1993; de Rudder et al., 2000; Klüsener et al., 2009). The *Sinorhizobium* type PmtA from *X. campestris* produces MMPE exclusively and is unable to further methylate it to DMPE and PC (Moser et al., 2014).

Most bacteria contain one Pmt enzyme for all three methylation steps but in some cases several Pmts with different specificities are required (Sohlenkamp et al., 2003). In the soybean symbiont *Bradyrhizobium japonicum*, PmtA methylates PE to MMPE, which serves as substrate for PmtX1-catalyzed methylation to DMPE and PC. *B. japonicum* encodes three further Pmt enzymes with distinct specificities (PmtX2-4), which are not expressed under standard laboratory conditions. PmtX1 and PmtX2 are similar to *R. sphaeroides* PmtA, whereas PmtA, PmtX3 and PmtX4 are homologous to *S. meliloti* PmtA (Minder et al., 2001; Hacker et al., 2008a,b). Like *B. japonicum*, *Rhizobium leguminosarum*, *Rhodopseudomonas palustris,* and *Rhizobium etli* seem to encode more than one *pmt* homolog (López-Lara et al., 2003; Martínez-Morales et al., 2003).

### THE PC SYNTHASE PATHWAY: A MEMBRANE-INTEGRATED ENZYME USES EXOGENOUS CHOLINE FOR PC SYNTHESIS

The second PC synthesis pathway in* A. tumefaciens* is catalyzed by the Pcs enzyme (**Figure 4**). Like *pmtA*, the *pcs* gene (*atu1793*) is located on the circular chromosome and is constitutively expressed (Wessel et al., 2006; Klüsener et al., 2009; Wilms et al., 2012). Pcs uses exogenous choline, which is transported via the high-affinity choline transport system ChoXWV. A *choXWV*-deficient strain is largely impaired in choline transport but can still produce PC when choline is present suggesting alternative choline uptake systems in *A. tumefaciens* (Aktas et al., 2011b). Similar to *A. tumefaciens*, the Pcs pathway in *S. meliloti* and *B. abortus* rely on exogenous choline delivered by a homologous Cho transport system (de Rudder et al., 1999; Dupont et al., 2004; Herrmann et al., 2013). Choline is a major component of eukaryotic membranes liberated by phospholipases from PC. Large amounts of free choline is found in homogenized plant tissues (Zeisel et al., 2003) and a recent study showed that considerable choline pools are also present on leaf surfaces. *Pseudomonas syringae* produces PC exclusively via the Pcs pathway and contains three choline transport systems with different specificities (Chen and Beattie, 2008). *P. syringae* exhibits chemotaxis towards choline and other quaternary amines. Extracellular choline is scavenged by *P. syringae* and enhances fitness during leaf colonization (Chen et al., 2013).

An *A. tumefaciens* *pcs* mutant produces PC via the remaining PmtA pathway and conversely PC production in a *pmtA* mutant depends on extracellular choline which might be delivered by the host plant. Only a *pmtA*/*pcs* double mutant lacks PC excluding alternative PC synthesis pathways in this organism (Wessel et al., 2006). Both *A. tumefaciens* PC biosynthesis pathways can be functionally reconstituted in *E. coli* demonstrating that PmtA and Pcs do not require *A. tumefaciens* specific cofactors or substrates (Klüsener et al., 2009).

The best-characterized Pcs enzyme derives from *S. meliloti* (de Rudder et al., 1999; Sohlenkamp et al., 2000; Solís-Oviedo et al., 2012). It catalyses the transfer of a phosphatidyl group from CDP-DAG to choline releasing a CMP molecule and PC. Enzyme activity depends on divalent cations like Mn^2^^+^ or Mg^2^^+^ and on detergents such as triton X100 (de Rudder et al., 1999). A topological study suggested that sinorhizobial Pcs is an integral membrane protein containing eight TM with N- and C- termini located in the cytosol. Pcs is a member of the CDP-alcohol phosphotransferase (CDP-OH-PT) protein superfamily containing a modified version of a conserved CDP-OH-PT motif (DX_2_DGX_2_ARX_12_GX_3_GX_3_D) characteristic for this enzyme family. Most of the conserved amino acids are located within a cytosolic loop connecting the TM domains II and III and are critical for enzyme activity as shown via mutagenesis (Solís-Oviedo et al., 2012; Geiger et al., 2013). Since the membrane-bound nature of Pcs enzymes has precluded their purification and biochemical characterisation, the precise reaction mechanism of Pcs enzymes is presently unknown but most likely proceeds via a sequential Bi–Bi reaction as in other CDP-OH-PT enzymes (Geiger et al., 2013).

It is not clear why two PC biosynthesis pathways operate simultaneously in *Agrobacterium* and some other bacteria. Although the Pcs pathway is energetically more favorable than the PE-methylation route, under conditions of choline limitation during competition with other bacteria, the Pmt pathway might be beneficial. In *Agrobacterium* both pathways seem to be constitutively present. PmtA activity is detected even in the presence of choline, when Pcs is active (Wessel et al., 2006; Klüsener et al., 2009). When two alternative PC synthesis pathways are present in eukaryotes, PC production via PE-methylation is repressed in the presence of choline used by the CDP-choline dependent pathway (Vance and Ridgway, 1988; Vance et al., 1997). It remains to be examined whether PmtA and Pcs pathways produce distinct PC pools with different fatty acyl chains as it is the case in eukaryotes (DeLong et al., 1999). Clearly, PC biosynthesis in *Agrobacterium* deserves further studies.

## NON-PHOSPHORUS LIPIDS AND BIOSYNTHETIC PATHWAYS

Since inorganic phosphate is limiting in most soils, bacteria have evolved exquisite strategies to deal with phosphate deficiency. One strategy is to partially replace membrane PLs by phosphorus-free lipids as shown for* S. meliloti*, *Pseudomonas fluorescens*, *R. sphaeroides,* and *A. tumefaciens*. Various phosphorus-free lipids appear in these organisms upon phosphate limitation such as sulfolipids, GLs, betaine lipids, or OLs (Benning et al., 1995; López-Lara et al., 2003; Geiger et al., 2010; Zavaleta-Pastor et al., 2010; Geske et al., 2013).

The *A. tumefaciens*-related α-proteobacterium *S. meliloti* has served as model system in this context. Its membranes are composed of the PLs PG, PE, MMPE, and PC when grown under phosphate-rich conditions. Phosphate limitation triggers the degradation of PE, MMPE, and PC and accumulation of the phosphate-lacking lipids DGTS-(*N*,*N*,*N*,-trimethyl)homoserine (DGTS), sulfoquinovosyl-DAG (SQD), and OLs. Phosphate-dependent membrane remodeling is regulated by the PhoR-PhoB system: under phosphate-limitation, the response regulator PhoB activates expression of genes responsible for OL and DGTS synthesis and for synthesis of an intracellular phospholipase C (PlcP). PlcP degrades the PLs PE, MMPE, and PC to the corresponding phosphoalcohols and DAG. Inorganic phosphate is released from the phosphoalcohols by yet unknown phosphatases and is used as a source for essential phosphate-dependent biological processes. The released DAG serves as substrate for the formation of the non-phosphorus lipids DGTS and SQD (Geiger et al., 1999; Zavaleta-Pastor et al., 2010).

**FIGURE 5:**
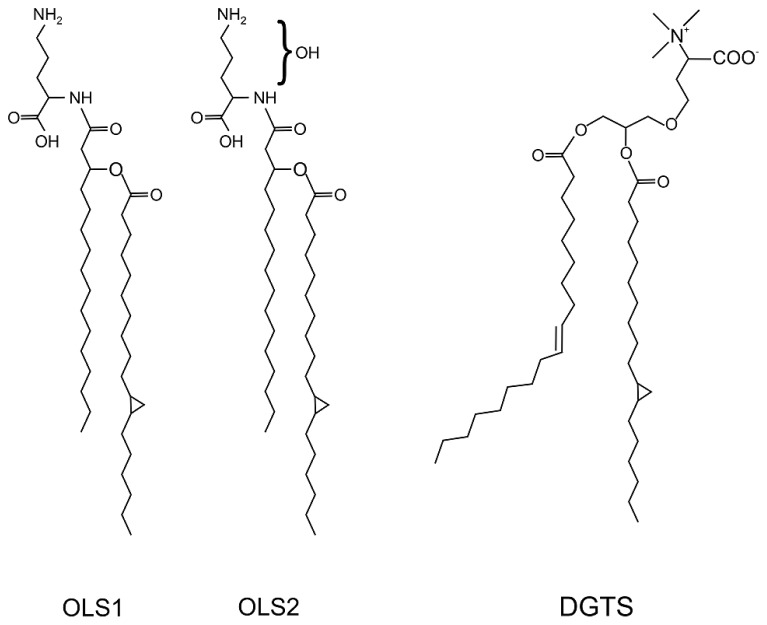
**Structure of the ornithine lipids OLS1/OLS2 and diacylglycerol trimethylhomoserine (DGTS) in *A. tumefaciens.*** The OLs contain C16 3OH and C19:0 cyclo fatty acids. OLS2 is hydroxylated within the ornithine moiety. DGTS contains 18:1 and 19:0 cyclo fatty acids (Geske et al., 2013; Vences-Guzmán et al., 2013).

Ornithine lipids are fatty-acylated amino acids free of phosphate and glycerol. The non-proteinogenic amino acid ornithine is connected via its α-amino group to a 3-hydroxy fatty acid and a second fatty acid chain is esterified to the 3-hydroxy group of the first fatty acid (**Figure 5**). OLs are widely distributed among eubacteria but absent from archaea and eukaryotes. Biosynthesis of OLs occurs via an acyl-ACP dependent two-step acylation of ornithine by two different acyltransferases. The first OL acyltransferases were discovered in *S. meliloti* (Weissenmayer et al., 2002; Gao et al., 2004). Acylation of ornithine occurs here via OlsB at the α-amino group to form lyso-ornithine (LOL), which in turn is acylated by OlsA at the 3-hydroxyl group to form OL (Gao et al., 2004; Geiger et al., 2013). Some bacteria modify their OLs by hydroxylation of the ornithine moiety or the ester- or amide-linked fatty acid. Three different OL hydroxylases are known in bacteria so far. OlsE homologs hydroxylate the ornithine moiety and the fatty acid portion is hydroxylated by OlsD (amide-linked) or OlsC (ester-linked) hydroxylases (Geiger et al., 2010; González-Silva et al., 2011; Vences-Guzmán et al., 2012). Several studies showed a contribution of hydroxylated OLs in microbe–host interactions and pH or thermal stress resistance (Rojas-Jiménez et al., 2005; González-Silva et al., 2011; Vences-Guzmán et al., 2011, 2012). It has been suggested that the additional hydroxyl groups increase the interaction between lipids via hydrogen bonds and thus, decrease the membrane fluidity and permeability, which might be advantageous under different stress conditions (Geiger et al., 2013). A recent study revealed a modification of OLs via methylation of the ornithine head group to mono-, di- and trimethyl-OL in planctomycetes isolated from an acidic and nutrient-poor ecosystem (Moore et al., 2013). Methylation of OLs increases their polarity and confers a more cylindrical shape, which possibly increases membrane stability similar to the bilayer forming lipid PC. Therefore, producing methyl-OLs might be an adaptation strategy to cope with acidity and nutrient scarcity in these organisms (Moore et al., 2013).

### TWO ORNITHINE LIPIDS ARE SYNTHESIZED IN *A. tumefaciens*

**FIGURE 6:**
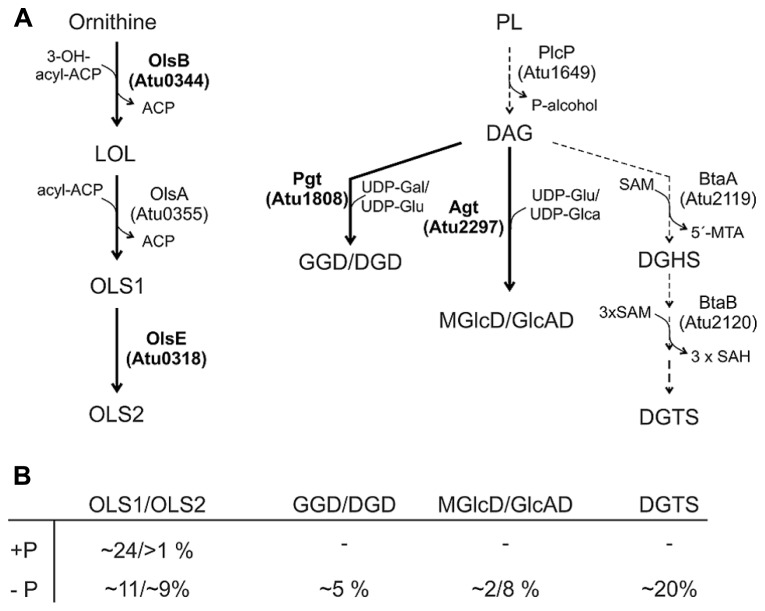
**Phosphorus-free lipid synthesis pathways in *A. tumefaciens.*** **(A)** Characterized pathways are indicated by thick arrows and enzymes in boldface letters. Putative biosynthetic pathways/enzymes are indicated with dashed arrows (Geske et al., 2013; ). For details see text. **(B)** Relative proportion of phosphorus-free lipids in *A. tumefaciens* C58C1 under phosphate-replete (+P) and phosphate depleted (-P) conditions (Geske et al., 2013). Gal, galactose; Glu, glucose; Glca, glucoronic acid.

In contrast to* S. meliloti* and* R. sphaeroides*, which produce only minor amounts of OLs under phosphate-replete conditions,* A. tumefaciens* accumulates significant amounts of two different OLs namely OLS1 and OLS2 even under full nutrient supply (Geske et al., 2013; Vences-Guzmán et al., 2013). In *A. tumefaciens* the OLs are composed of the fatty acids C16 3OH and C19:0 cyclo as shown by mass spectrometry analyses. OLS2 is the hydroxylated form of OLS1 containing the hydroxyl group within the ornithine moiety (Geske et al., 2013; Vences-Guzmán et al., 2013; **Figure 5**). *Agrobacterium* encodes the three *ols* homologs *olsA* (*atu0355*), *olsB* (*atu0344*), and *olsE* (*atu0318*) on the circular chromosome (Vences-Guzmán et al., 2012). *olsE* and *olsB* mutants in the *A. tumefaciens* A208 strain revealed that *olsB* is essential for formation of both OLS1 and OLS2 whereas *olsE* is only required for OLS2 synthesis (**Figure 6**). Heterologous expression of *olsE* resulted in OLS2 formation providing further evidence that OlsE is the hydroxylase responsible for OLS2 formation. Thus, the first step in OL synthesis in *Agrobacterium* is mediated by the acyltransferase OlsB forming ornithine to the lyso-ornithine lipid (LOL). Subsequently, LOL might be acylated via the putative OlsA to form OLS1. OLS2 formation is completed by hydroxylation of the ornithine moiety by OlsE (Vences-Guzmán et al., 2013).

Under low phosphate conditions, both OLs accumulate to a total amount of 45–50% whereas the total PL content decreases in *A. tumefaciens* A208. A putative Pho box is located in the promoter region of *olsB* suggesting PhoB-induced expression under phosphate starvation (Geske et al., 2013; Vences-Guzmán et al., 2013). In the *olsB* mutant, lack of OLs seems to be compensated by an increase in DGTS and GL accumulation under phosphate reduced conditions (Vences-Guzmán et al., 2013).

Ornithine lipids production under phosphate starvation conditions seems to vary in different *Agrobacterium* strains. In contrast to *A. tumefaciens* A208, the total amount of OLs does not change under phosphate-limiting conditions in *A. tumefaciens* C58C1 but the degree of hydroxylation is ninefold increased (Geske et al., 2013; Vences-Guzmán et al., 2013). Deviations in the experimental setups such as the growth media and the precise phosphate concentrations might account for these differences.

### *A. tumefaciens* PRODUCES FOUR DIFFERENT GLYCOLIPIDS AND A BETAINE LIPID UNDER PHOSPHATE DEPRIVATION

Glycolipids contain carbohydrate residues, which are glycosidically bound to the 3-position of a *sn*-1,2-DAG (Shaw, 1970). Different GLs are produced in bacteria under phosphate starvation. The photosynthetic bacterium *R. sphaeroides* produces the unique GL glucosylgalactosyl-DAG (GGD) with α-glucose (1→4)-linked to β-galactose (Benning et al., 1995). A series of GLs found in the nitrogen-fixing symbiont *Mesorhizobium loti* differs from the rhodobacterial GL. *M. loti* produces the GLs GGD, digalactosyl-DAG (DGD), and different molecular species of triglycosyl-DAG with various combinations of galactose and glucose in the head. All of the sugars are in β-configuration and (1→6)-linked to each other. Additionally, *M. loti* contains two further GLs with yet unknown head groups (Devers et al., 2011).

*A. tumefaciens* produces under phosphate deprivation four different GLs and DGTS accounting to 35% of the total lipids (Geske et al., 2013). The GLs have been identified recently as GGD and DGD with a β-configuration and monoglucosyl-DAG (MGlcD) and glucuronosyl-DAG (GlcAD) with a α-configuration (Geske et al., 2013; Semeniuk et al., 2014). The relative amount of these lipids in *A. tumefaciens* C58C1 is given in **Figure 6B**. Similar to *M. loti*, GGD and DGD are synthesized in *A. tumefaciens* via a processive glycosyltransferase namely Pgt (**Figure 6**) by a successive transfer of glucosyl and/or galactosyl residues to DAG. Functional characterisation of Pgt in *E. coli* and *Pichia pastoris* and overexpression in *Agrobacterium* revealed a broad substrate specificity concerning the glycosyl acceptor (DAG or ceramides) and sugar residues (uridine diphospho, UDP-galactose or UDP-glucose). However, Pgt favors DAG over ceramide and UDP-galactose over UDP-glucose (Hölzl et al., 2005). The promoter region of *pgt* contains a predicted Pho box suggesting an induced Pgt synthesis upon phosphate limitation mediated via the PhoR-PhoB system. A* pgt* mutant lacks GGD and DGD but the remaining lipids accumulate wild type-like (Geske et al., 2013).

Synthesis of MGlcD and the acidic GlcAD in *A. tumefaciens* is catalyzed by a single promiscuous glycosyltransferase namely Agt encoded by *atu2297* (**Figure 6**). Enzyme assays with recombinant Agt in *E. coli* protein extracts provided evidence that Agt uses UDP-glucose and UDP-glucuronic acid as sugar donors for MGlcD and GlcAD synthesis, repectively (**Figure 6**; Semeniuk et al., 2014). *A. tumefaciens* Agt is the first described glycosyltransferase using sugars with different chemistry. An *A. tumefaciens* *agt* mutant is deficient in MGlcD and GlcAD formation and loss of these GLs is compensated by a twofold increase in GGD and DGD. Remarkably, while DGTS and all other PLs are not influenced in the* agt* mutant, PC amount is strongly reduced. Deletion of both *pgt* and* agt* genes results in the loss of all GLs, which is compensated by a strong DGTS accumulation. Similar to the single *agt* mutant, the PC content of the double mutant is strongly reduced. One reason might be that PC is degraded to provide DAG for GGD/DGD synthesis in case of the *agt* mutant or for DGTS synthesis in the double mutant. It is unclear, however, why specifically PC and no other PL is turned over to supply DAG for the synthesis of phosphate-free lipids. Another reason might be that reduction of the bilayer-stabilizing PC in membranes missing the acidic GlcAD is necessary to sustain membrane structure and fluidity (Semeniuk et al., 2014). In *S. meliloti*, loss of the acidic glycolipid SQD is compensated by an increase of the anionic and bilayer-forming lipid PG (Weissenmayer et al., 2000).

Since loss of all GLs has no impact on growth and virulence even under phosphate-limited conditions, *A. tumefaciens* seems to compensate the lack of all GLs by DGTS (Geske et al., 2013; Semeniuk et al., 2014). A *S. meliloti* mutant deficient in all phosphate-free lipids shows decreased growth under phosphate starvation but is not influenced in nodule formation on its host alfalfa (López-Lara et al., 2005) suggesting that these lipids function as bulk membrane lipids. Whether lack of all GLs and DGTS impacts *A. tumefaciens* physiology and virulence remains to be seen.

The acidic GlcAD in *A. tumefaciens* might be the counterpart of the glycolipid SQD which is absent in *Agrobacterium* but widespread in photosynthetic organisms and present in a few non-photosynthetic bacteria such as some rhizobia (López-Lara et al., 2003). The role of SQD in these organisms is still unclear. It has been speculated that SQD might have a special role in photosynthesis or is required for nodule formation and nitrogen fixation. However, SQD-free mutants of the photosynthetic purple bacterium *R. sphaeroides* and the nitrogen fixing *S. meliloti* are not compromised in photosynthesis and symbiosis, respectively suggesting no general function of bacterial SQD in these processes (Benning et al., 1993; Weissenmayer et al., 2002; López-Lara et al., 2003).

DGTS-(*N*,*N*,*N*,-trimethyl)homoserine is a betaine-ether linked glycerolipid abundant in membranes of plants, algae, and fungi and is found in a few bacteria (Dembitsky, 1996; López-Lara et al., 2003). In *Agrobacterium* membranes DGTS is a major non-phosphorus lipid (~20 mol%) during phosphate starvation (**Figure 6B**). Similar to PC, DGTS is a zwitterionic lipid containing a quaternary amino head group (**Figure 5**). It has been observed that the content of PC and DGTS within a cell is reciprocal. Organisms containing major amounts of PC produce only traces of DGTS and vice versa (Geiger et al., 2010). The structural similarity and the inverse relationship between DGTS and PC concentrations led to the speculation that these two lipids are functionally interchangeable (López-Lara et al., 2003; Geiger et al., 2010; Devers et al., 2011).

DGTS-(*N*,*N*,*N*,-trimethyl)homoserine synthesis in *R. sphae-*roides and *S. meliloti* occurs via the BtaA/B system (Klug and Benning, 2001; López-Lara et al., 2005). BtaA is a SAM/DAG 3-amino-3-carboxypropyl transferase that converts DAG to DAG-homoserine (DGHS) using SAM as homoseryl donor. Subsequently, DGHS is threefold methylated via BtaB, a SAM:DAG-homoserine-*N*-methyltransferase, to DGTS. Expression of the sinorhizobial *btaA* and *btaB* genes is PhoB regulated. BtaA (*atu2119*) and BtaB (*atu2120*) homologs which have not been characterized yet are encoded in the *A. tumefaciens* genome suggesting a similar DGTS biosynthesis and regulation (Yuan et al., 2006; **Figure 6**). In *A. tumefaciens*, DGTS and GL accumulation under phosphate limitation also seems to be controlled not only on transcriptional level of the responsible biosynthesis genes but also via DAG substrate availability. A PlcP homolog, encoded by *atu1649* in the *A. tumefaciens* genome (Geske et al., 2013) suggests a similar membrane remodeling mechanism as described in *S. meliloti* (Zavaleta-Pastor et al., 2010; Geiger et al., 2013). Interestingly, phosphate starvation results not only in the replacement of PLs by non-phosphorus lipids in *A. tumefaciens* but also in changes in fatty acid composition of DAG and PLs with a shift from 18:1 to 19:0 cyclo fatty acids (Geske et al., 2013). Whereas under full nutrition PLs are mainly composed of 18:1 (50–60%) fatty acids and contain low proportions of 19:0 cyclo (20 and 40% in PC) fatty acid, phosphate limitation results in a decrease in 18:1 (10%) and a strong increase in 19:0 cyclo (60%) fatty acids (Geske et al., 2013). *A. tumefaciens* codes for a putative cyclopropane fatty acid (CFA) synthase presumably responsible for this modification (Geske et al., 2013). Cyclopropanation of pre-existing unsaturated fatty acids is widespread in bacteria and maximal activity is observed during stationary phase. The biological role of CFA containing lipids in bacteria is not fully understood. Accumulation of CFAs in *E. coli* is correlated with acid tolerance and seems to be important for pathogenic bacteria–host interactions as shown for *Mycobacterium tuberculosis* (Chang and Cronan, 1999; Glickman et al., 2000; Zhang and Rock, 2009). A twofold increase in CFA content under phosphate starvation and acid conditions is also observed in *S. meliloti*. Here, two CFA synthases have been described, with Cfa1 essential for cyclopropanation of fatty acids under tested conditions. Both* cfa* genes are not required for symbiotic nitrogen fixation in *S. meliloti* (Saborido Basconcillo et al., 2009). Whether cyclopropanated lipids are required for *A. tumefaciens* virulence remains to be determined.

## IMPORTANCE OF MEMBRANE LIPIDS FOR *A. tumefaciens* PHYSIOLOGY AND PATHOGENESIS

### PHOSPHATIDYLCHOLINE IS CRUCIAL FOR AGROBACTERIUM VIRULENCE

**FIGURE 7:**
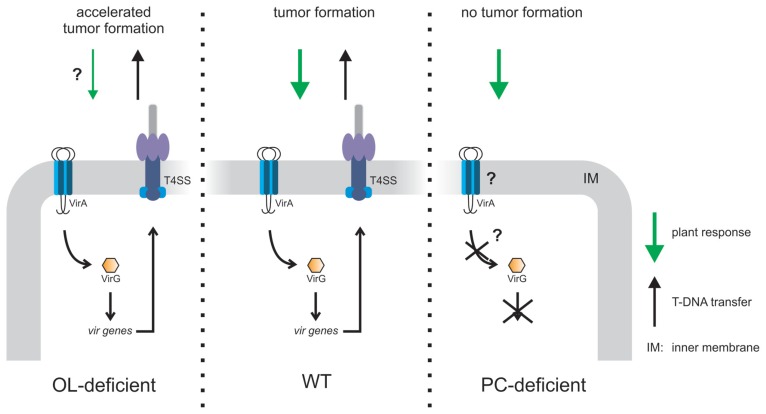
**Model of PC- or OL-dependent effects on *A. tumefaciens* infection efficiency.** OL-lacking *A. tumefaciens* induces accelerated tumor formation on potato disks compared to wild type (WT) probably due to reduced plant defense (Vences-Guzmán et al., 2013). A PC-deficient mutant is unable to elicit tumors on *Kalanchoë* leaves since VirA/G controlled *vir* gene expression is impaired. As a consequence, the type4 secretion system (T4SS) is not produced which is essential for tumor formation (Wessel et al., 2006; Klüsener et al., 2010).

Although the typical eukaryotic membrane lipid PC is rarely found in bacteria it is a main constituent of *A. tumefaciens* inner and outer membranes suggesting an important role for this organism (Klüsener et al., 2009). Indeed, loss of PC causes different physiological defects. A PC-deficient mutant is impaired in growth on solid medium at elevated temperatures and is unable to grow in the presence of the anionic detergent SDS. Furthermore, it is less motile and produces larger amounts of surface-attached biomass (Klüsener et al., 2009). The motility defect is explained by reduced flagellar proteins (FlaA and FlaB) in minimal medium (Klüsener et al., 2009, 2010). The most striking phenotype of a PC-deficient mutant is its defect in tumor formation due to loss of the VirB/D4 Type 4 SS (T4SS) essential for T-DNA transfer (Wessel et al., 2006). In response to plant stimuli the two component system VirA/G controls the expression of 11 transcriptional units, among them the *virB* and *virD* operons encoding the T4SS. The homodimeric histidine kinase VirA is anchored in the inner membrane. Plant-released signals, e.g., phenolic compounds are recognized by a cytoplasmic linker domain whereas acidic pH and monosaccharides are perceived by the periplasmic domain (Nair et al., 2011). The global response to PC-deficiency in *A. tumefaciens* as determined by proteomics and transcriptomics shows that the VirA/G-controlled *vir* gene expression under virulence-induced conditions is drastically reduced thus explaining the absence of the T4SS (Klüsener et al., 2010). Only a limited set of other genes coding for membrane-related proteins were changed in the absence of PC. Expression of *virG* in the PC-deficient mutant was also dramatically reduced suggesting that lack of virulence gene induction is due to low *virG* expression. Since the loss of *vir* gene expression in a PC-deficient mutant cannot be complemented by expression of a plasmid-encoded wild type *virG* but by a constitutively active VirG, it seems that a non-functional VirA sensor kinase is responsible for the loss of virulence gene expression in the PC-lacking *Agrobacterium* mutant. These observations suggest that signal transduction between VirA and VirG is impaired in the absence of PC, possibly due to limitations in membrane insertion or folding of VirA (**Figure 7**). It remains to be seen whether the observed phenotypic defects in the PC-deficient mutant are PC-specific or a consequence of altered bulk physico-chemical properties of the membrane in the absence of PC. The structural organization of membranes is defined by the physical properties and shape of membrane lipids. Cylindrical-shaped lipids such as PG or PC are bilayer-forming lipids whereas cone-shaped lipids such as PE are considered non-bilayer forming lipids (van Meer et al., 2008). However, non-bilayer lipids can form bilayer-structures depending on solvent conditions, alkyl chain composition, and temperature.

We do not know yet whether loss of other PLs such as PE, PG, or CL *in A. tumefaciens* causes similar effects on physiology and VirA/G-mediated signal transduction. It has been shown that PE can act as molecular chaperone for proper folding and function of membrane proteins such as the lactose permease LacY in *E. coli* (Bogdanov et al., 1999; Bogdanov and Dowhan, 2012). Replacement of PE by PC during reconstitution of the ABC multidrug exporter HorA from *Lactobacillus* *brevis* into membrane vesicles altered the orientation of TM helices and abolished transport function (Gustot et al., 2010). The effect of PC depletion on membrane proteins (Klüsener et al., 2010) suggests that PC and probably other PLs play a role in membrane protein homeostasis in *A. tumefaciens*.

It is important to note that the requirement of PC for productive host–microbe interactions is not restricted to *A. tumefaciens*. PC-deficient *S. meliloti* mutants are unable to establish nitrogen-fixing symbiosis with their host plant alfalfa (Sohlenkamp et al., 2003). Reduced PC levels in *B. japonicum*, the symbiont of the soybean *Glycine max* cause formation of nodules with impaired nitrogen fixation activity (Minder et al., 2001). A PC-deficient mutant of the intracellular human pathogen *Legionella pneumophila* shows lowered cytotoxicity and adhesion to the host cell. Loss of PC affects the Dot/Icm T4SS, system which delivers virulence factors into the cytosol of infected cells and is required for intracellular growth (Conover et al., 2008). In *P. syringae* PC is essential for secretion of the HrpZ harpin effector protein possibly due to a non-functional T3SS (Xiong et al., 2014). *B. abortus*, the causative agent of brucellosis produces PC via the Pcs pathway. A *pcs* mutant is defective in PC formation and attenuated in virulence when assayed in the mouse model (Comerci et al., 2006). PC is not generally critical for physiology or microbe–host interactions. Loss of PC in the opportunistic pathogen *P*.* aeruginosa* did not affect physiology and virulence (Malek et al., 2012). It is important to note here that PC is only a minor (~4%) component of *P. aeruginosa* membranes (Geiger et al., 2013).**

### LACK OF THE HYDROXYLATED ORNITHINE LIPID OLS2 IN *A. tumefaciens* CAUSES ACCELERATED TUMOR FORMATION

Although various bacteria deficient in OL biosynthesis have been characterized, the function of OLs still is largely unclear. OLs have been implicated in high-temperature tolerance in *Burkholderia cepacia* (Taylor et al., 1998). In *Bordetella pertussis* and *Flavobacterium meningosepticum* OLs are involved in hemagglutination and stimulation of macrophages (Kawai and Yano, 1983; Kawai and Akagawa, 1989; Kawai et al., 1999). In *Rhodobacter capsulatus* OL is critical for optimal yields of cytochrome c (Aygun-Sunar et al., 2006). In Gram-negative bacteria OLs are enriched in the outer membrane. It has been postulated that the zwitterionic OLs increase outer membrane stability via stabilization of the negative charges of LPS. Hydroxylation of OLs often correlates with bacterial stress response (Vences-Guzmán et al., 2012). It is speculated that the additional OH group increases hydrogen bonding between the lipid molecules as shown for the 2-hydroxylated lipid A in *Salmonella typhimurium*. This would decrease the membrane fluidity and make it less permeable (Gibbons et al., 2000; Vences-Guzmán et al., 2012).

In *A. tumefaciens* A208 grown at low temperatures (15°C) unmodified OLS1 is completely hydroxylated to OLS2 suggesting a role of this modified OL in temperature stress. However, lack of both OLs has no impact on growth even under high osmolarity or at low temperature. Interestingly, *A. tumefaciens* A208 mutants devoid of OLS2 induce about 1 week earlier tumors and consequently, the tumor size is increased compared to wild type induced tumors (Vences-Guzmán et al., 2013). OLs share a 3-acyl-oxyacylamide structure with lipid A of Gram-negative bacteria, which is an elicitor in plant–microbe interactions (Scheidle et al., 2005; Silipo et al., 2008; Madala et al., 2011). It has been speculated that hydroxylated OLs cause a plant defense response, which might be lowered in the absence of OLs thus explaining accelerated tumor formation (**Figure 7**).

The role of OLs in plant interaction cannot be generalized and it seems that OLs have different functions in different bacteria. In contrast to *A. tumefaciens*, the two modified OLs P1 and P2 are necessary for a successful symbiotic interaction in the nitrogen-fixing symbiont *R. tropici* CIAT899, which is highly tolerant to different environmental stresses (Rojas-Jiménez et al., 2005). The OL in *S. meliloti* is required for normal growth under phosphate-limiting conditions but not necessary for symbiotic performance (López-Lara et al., 2005).

## CONCLUSION

Recent progress in lipid analysis technologies has revealed a surprising diversity in bacterial membrane lipid biosynthesis. The membrane composition is very dynamic and substantially remodeled in response to environmental changes. A future challenge will be to define the physiological role of specific lipids at the molecular level. The phenotypic characterisation of lipid biosynthesis mutants has already provided interesting insights into the *in vivo* function of various lipids but has considerable limitations. Sometimes it is difficult to interpret whether the observed phenotypes are direct or indirect because most bacteria are able to compensate the loss of one lipid by changing the overall lipid composition. One interesting model organism in this context is *A. tumefaciens*, the natural genetic engineer of plants. Two specific membrane lipids, the PL PC and a phosphate-free lipid OL affect virulence with opposing outcomes. PC-deficiency causes a loss of virulence gene expression and tumor formation whereas lack of OLS2 accelerates tumorigenesis. Biophysical and biochemical studies combined with genetic manipulation are needed to understand the precise molecular mechanisms, by which these lipids influence membrane properties and *Agrobacterium-*mediated tumor formation.

## Conflict of Interest Statement

The authors declare that the research was conducted in the absence of any commercial or financial relationships that could be construed as a potential conflict of interest.
